# Generating normal networks via leaf insertion and nearest neighbor interchange

**DOI:** 10.1186/s12859-019-3209-3

**Published:** 2019-12-17

**Authors:** Louxin Zhang

**Affiliations:** Department of Mathematics, National University of Singapore, 10 Lower Kent Ridge Road, Singapore, 119076 Singapore

**Keywords:** Tree-child networks, normal networks

## Abstract

**Background:**

Galled trees are studied as a recombination model in theoretical population genetics. This class of phylogenetic networks has been generalized to tree-child networks and other network classes by relaxing a structural condition imposed on galled trees. Although these networks are simple, their topological structures have yet to be fully understood.

**Results:**

It is well-known that all phylogenetic trees on *n* taxa can be generated by the insertion of the *n*-th taxa to each edge of all the phylogenetic trees on *n*−1 taxa. We prove that all tree-child (resp. normal) networks with *k* reticulate nodes on *n* taxa can be uniquely generated via three operations from all the tree-child (resp. normal) networks with *k*−1 or *k* reticulate nodes on *n*−1 taxa. Applying this result to counting rooted phylogenetic networks, we show that there are exactly $\frac {(2n)!}{2^{n} (n-1)!}-2^{n-1} n!$ binary phylogenetic networks with one reticulate node on *n* taxa.

**Conclusions:**

The work makes two contributions to understand normal networks. One is a generalization of an enumeration procedure for phylogenetic trees into one for normal networks. Another is simple formulas for counting normal networks and phylogenetic networks that have only one reticulate node.

## Background

Phylogenetic networks have been used to date both vertical and horizontal genetic transfers in evolutionary genomics and population genetics in the past two decades [1–3]. A rooted phylogenetic network (RPN) is a directed acyclic digraph in which all the sink nodes are of indegree 1 and a unique source node is designated as the root, where the former represent a set of taxa (e.g, species, genes, or individuals in a population) and the latter represents the least common ancestor of the taxa. Moreover, the other nodes in a RPN are divided into tree nodes and reticulate nodes, where reticulate nodes represent reticulate evolutionary events such as horizontal genetic transfers and genetic recombination.

The topological properties of RPNs are much more complicated than phylogenetic trees [2, 4, 5]. Therefore, different mathematical issues arise in the study of RPNs. First, phylogenetic reconstruction problems are often NP-hard even for trees [6, 7]. As such, a phylogenetic reconstruction method often uses nearest neighbor interchanges (NNIs) or other rearrangement operations to search for an optimal tree or network [8, 9]. Recently, different variants of NNI have been proposed for RPNs [10–16].

Second, to develop efficient algorithms for NP-complete problems on RPNs, simple classes of RPNs have been introduced, including galled trees [17, 18], tree-child networks (TCNs) [19], normal networks [20], reticulation-visible networks [4] and tree-based networks [21, 22] (see also [5, 23]). For instance, a RPN is a TCN if every non-leaf node has a child that is a tree node or a leaf. Although these network classes have been intensively investigated, their topological structures remain unclear [5, 24]. How to efficiently enumerate and count normal networks remains unclear [25–30].

This work makes two contributions to understanding TCNs and normal networks. It is a well-known fact that all phylogenetic trees on *n* taxa can be generated by inserting the *n*-th taxa in every edge of all the phylogenetic trees on *n*−1 taxa. We prove that all TCNs with *k* reticulate nodes on *n* taxa can be uniquely generated via three operations from TCNs with *k*−1 or *k* reticu- late nodes on *n*−1 taxa (Theorem 1, “Generating TCNs and normal networks” section). Using this fact, we obtain recurrence formulas for counting TCNs and normal networks (“Counting formulas” section). In particular, simple formulas are given for the number of RPNs and normal networks with one reticulate node, respectively.

## Methods

### Basic notation

A RPN over a finite set of taxa *X* is an acyclic digraph such that:

- there is a unique node of indegree 0 and outdegree 1, called the *root*;
- there are exactly |*X*| nodes of outdegree 0 and indegree 1, called the *leaves* of the RPN, each being labeled with a unique taxon in *X*;
- each non-leaf/non-root node is either a *reticulate node* that is of indegree 2 and outdegree 1, or a *tree node* that is of indegree 1 and outdegree 2; and
- there are no parallel edges between a pair of nodes.

**Fig. 1:**
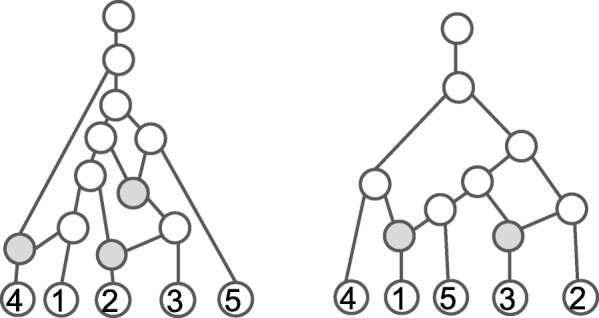
Two tree-child networks on {1,2,3,4,5}, where reticulate and tree nodes are drawn as filled and unfilled circles, respectively. Only the right network is normal. Here, edge downward orientation is omitted

Two RPNs are drawn in Fig. 1, where each edge is directed away from the root and both the root and edge orientation are omitted. For a RPN *N*, we use ${\mathcal {V}}(N), {\mathcal {R}}(N), {\mathcal {T}}(N)$ and ${\mathcal {E}}(N)$ to denote the set of all nodes, the set of reticulate nodes and the set of tree nodes and the set of directed edges for *N*, respectively.

Let $u\in {\mathcal {V}}(N)$ and $v\in {\mathcal {V}}(N)$. The node *u* is said to be a *parent* (resp. a *child*) of *v* if $(u, v)\in {\mathcal {E}}(N)$ (resp. $(v, u)\in {\mathcal {E}}(N)$). Every reticulate node *r* has a unique child, named *c*(*r*), whereas every tree node *t* has a unique parent, named *p*(*t*). Furthermore, *u* is an *ancestor* of *v* or, equivalently, *v* is *belowu* if there is a direct path from the network root to *v* that contains *u*. We say that *u* and *v* are *incomparable* if neither of them is an ancestor of the other.

Let $e=(u, v)\in {\mathcal {E}}(N)$. It is a *reticulate edge* if *v* is a reticulate node and a *tree edge* otherwise. Hence, a tree edge leads to either a tree node or a leaf.

A *phylogenetic tree* is simply a RPN with no reticulate nodes.

A *TCN* is a RPN in which every non-leaf node has a child that is a tree node or a leaf or, equivalently, there is a path from every non-leaf node to some leaf that consists only of tree edges. Both RPNs in Fig. 1 are tree-child.

A *normal* network is a TCN in which every reticulate node satisfies the following condition:

(**The normal condition**) The two parents are incomparable.

The first PRN in Fig. 1 is not normal, as a parent of the left most reticulate node is an ancestor of the other in the network.

### Generating TCNs and normal networks

**Fig. 2:**
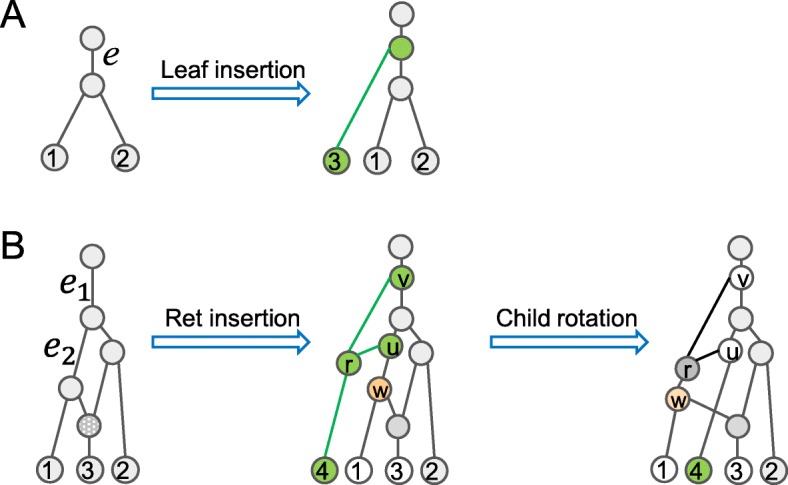
Insertion and child rotations for tree-child networks. **a** Leaf 3 is attached to a tree edge. **b** The reticulation insertion is applied to attach a new reticulate node *r* onto two tree edges. The child rotation swaps the tree node *w* (yellow) and Leaf 4. Here, green nodes and edges are added nodes
- **Reticulation insertion** For a pair of tree edges *e*_1_=(*u*_1_,*v*_1_) and *e*_2_=(*u*_2_,*v*_2_) of *N*, which are not necessarily distinct, insert a new node *w*_1_ to subdivide *e*_1_ and a new node *w*_2_ to subdivide *e*_2_, attach a new reticulate node *r* as the common child of *w*_1_ and *w*_2_ and make Leaf (*n*+1) to be the child of *r*. In this case, we say that *rstraddles* *e*_1_ and *e*_2_. We use Ret-Insert(*N*,*e*_1_,*e*_2_,*n*+1) to denote the resulting network. We simply write Ret-Insert(*N*,*e*,*n*+1) if *e*_1_=*e*_2_=*e*.
- **Child rotation** Let *r* be a reticulate node with parents $u \in {\mathcal {T}}(N)$ and *v*. If *u* is not an ancestor of *v*, exchange the unique child of *r* and the other child of *u*. The resulting network is denoted by C-Rotate(*N*,*u*,*r*).

We define the following rearrangement operations for TCNs *N* on [1,*n*], which are illustrated in Fig. 2:

- **Leaf insertion** For a tree edge $e=(u, v)\in {\mathcal {E}}(N)$, insert a new node *w* to subdivide *e* and attach Leaf *n*+1 below *w* as its child. The resulting network is denoted by Leaf-Insert(*N*,*e*,*n*+1), in which *w* is a tree node.

Note that a child rotation is a special case of the rNNI rearrangement introduced by Gambette et al. in [12]. Let ${\mathcal {T}CN}_{k}(n)$ denote the set of TCNs with *k* reticulations on [1,*n*].

#### **Proposition 1**

Let $M\in {\mathcal {T}CN}_{k}(n)$ and let *e*_1_ and *e*_2_ be two tree edges of *M*. Then,

$$\begin{array}{@{}rcl@{}} \text{Ret-Insert}\left(M, e_{1}, e_{2}, n+1\right)\in {\mathcal{T}CN}_{k+1}(n+1),\\ \text{Ret-Insert}(M, e_{1}, n+1)\in {\mathcal{T}CN}_{k+1}(n+1). \end{array} $$

#### *Proof*

The second statement is a special case of the first. Let *e*_1_=(*u*,*v*) and *e*_2_=(*x*,*y*). Since *e*_1_ and *e*_2_ are tree edges, both *v* and *y* are tree nodes or leaves. Let *r* be the added reticulate node. Then the parents of *r* have *v* and *y* as their child, respectively, the nodes *u* and *x* have the parents of *r* as their tree node child; Leaf *n*+1 is the tree child *r*. Additionally, all the other nodes have the same children as in *M*. Therefore, Ret-Insert(*M*,*e*_1_,*e*_2_,*n*+1) is a TCN. □

#### **Proposition 2**

Let $M\in {\mathcal {T}CN}_{k+1}(n+1)$. Assume that $r\in {\mathcal {R}}(M)$ and its parents are *u* and *v* such that *u* is not an ancestor of *v* in *M*. Then,

$$\begin{array}{@{}rcl@{}} \text{C-Rotate}\left(M, u, r \right)\in {\mathcal{T}CN}_{k+1}(n+1). \end{array} $$

#### *Proof*

Let *M*^′^=C-Rotate(*M*,*u*,*r*). Since *u* is a parent of *r* and *M* is tree-child, *u* is a tree node. Let *w* be the other child of *u* and let *z* be the unique child of *r*. Since *M* is tree-child, *z* and *w* are tree nodes (see Fig. 2). The tree node *z* becomes the child of *u* Therefore, every node also has a child that is a tree node or a leaf in *M*^′^. □

By definition, *w* becomes a child of *r* and *z* becomes a tree node child of *u* in *M*^′^. If *M*^′^ contains a directed cycle *C*, *C* must contain *v* and *w*, implying that *u* is an ancestor of *v* in *M*, a contradiction. Therefore, *M*^′^ is acyclic and $M' \in {\mathcal {T}CN}_{k+1}(n+1)$.

#### **Proposition 3**

Let $N\in {\mathcal {T}CN}_{k+1}(n+1)$.

(i) If Leaf (*n*+1) is the child of a reticulate node *r*, *N* can then be obtained from an $M\in {\mathcal {T}CN}_{k}(n)$ via a reticulation insertion.

(ii) If Leaf (*n*+1) is the child of a tree node *t* and the sibling of *n*+1 is also a tree node, *N* can then obtained from an $M\in {\mathcal {T}CN}_{k+1}(n)$ via a leaf insertion.

(iii) If Leaf (*n*+1) is a child of a tree node *t* and the sibling of *n*+1 is a reticulate node, *N* can then be obtained from an $M\in {\mathcal {T}CN}_{k+1}(n+1)$ via a child rotation.

#### *Proof*

(i) Let *r* have parents *u*_1_ and *u*_2_ in *N*. Since *N* is a TCN, *u*_1_ and *u*_2_ are tree nodes and so are their children other than *r*. Let *w*_*i*_ and *v*_*i*_ be the parent and the child of *u*_*i*_ such that *v*_*i*_≠*r*, respectively, for each *i*=1,2. Since *r* is a reticulate node, *v*_1_ and *v*_2_ are tree nodes. Without loss of generality, we assume that *u*_2_ is not the parent of *u*_1_. There are two cases for consideration.

**Fig. 3:**
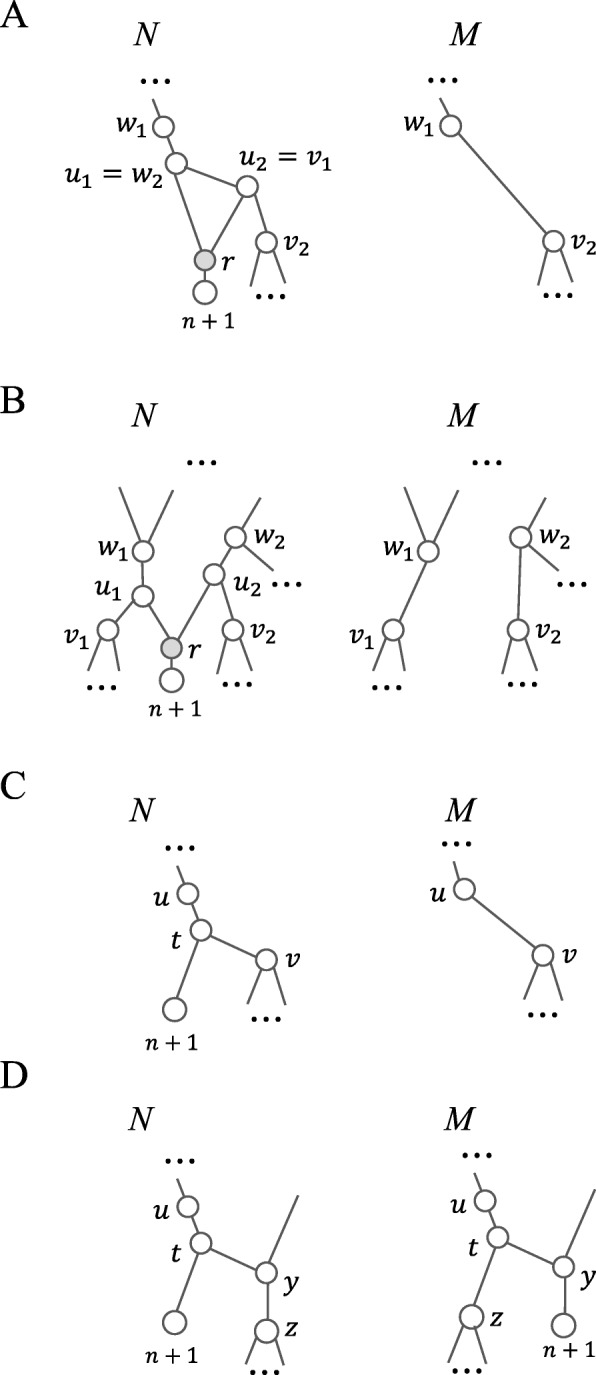
An illustration of the proof of Proposition 3. **a** The reticulate node parent *r* of *n*+1 has two adjacent parents. **b** The reticulate node parent *r* of *n*+1 has two non-adjacent parents. **c** The parent and sibling of *n*+1 are both a tree node. **d** The parent of *n*+1 is a tree node, whereas the sibling of *n*+1 is a reticulate node

If *u*_1_ is the parent of *u*_2_, then *u*_1_=*w*_2_ and *u*_2_=*v*_1_ (Fig. 3a). Removing Leaf (*n*+1),*u*_1_ and *u*_2_ (together with incident edges) and adding an edge *e*=(*w*_1_,*v*_2_) produce a TCN *M* with *k* reticulations such that *N*=Ret-Insert(*M*,*e*,*n*+1).

If *u*_1_ is not the parent of *u*_2_, then, *w*_1_≠*u*_2_ (Fig. 3b). After removing Leaf *n*+1,*u*_1_ and *u*_2_ (together with incident edges) and adding two edges *e*_*i*_=(*w*_*i*_,*v*_*i*_) (*i*=1,2), we obtain a TCN *M* such that *N*=Ret-Insert(*M*,*e*_1_,*e*_2_,*n*+1).

(ii) Let *u* be the parent of *t* and let *v* be the sibling of Leaf *n*+1 (Fig. 3c). By assumption, *v* is a tree node. After removing *t* and Leaf (*n*+1) (together with incident edges) and adding *e*=(*u*,*v*), we obtain a TCN $M\in {\mathcal {T}CN}_{k+1}(n)$ such that *N*=Leaf-Insert(*M*,(*u*,*v*),*n*+1).

(iii) Let *y* be the sibling of *n*+1 that is a reticulate node (Fig. 3d). Let *z* be the child of *y* and let *M*=C-Rotate(*N*,*t*,*y*). Since *z* is below *y* and *y* is below *t* in *N*, neither attaching the tree node *z* below *t* nor attaching Leaf (*n*+1) below *y* generates a directed cycle in *M*. Hence, $M\in {\mathcal {T}CN}_{k+1}(n+1)$ in which Leaf (*n*+1) is the child of a reticulate node *y* such that *N*=C-Rotate(*M*,*t*,*y*). □

#### **Proposition 4**

Let $N_{1}, N_{2} \in {\mathcal {T}CN}_{k}(n)$.

(i) Leaf-Insert(*N*_1_,*e*_1_,*n*+1) is identical to Leaf-Insert(*N*_2_,*e*_2_,*n*+1) iff *N*_1_=*N*_2_ and *e*_1_=*e*_2_.

(ii) Ret-Insert(*N*_1_,*e*_1_,*e*1′,*n*+1) is identical to Ret-Insert(*N*_2_,*e*_2_,*e*2′,*n*+1) iff *N*_1_=*N*_2_.

(iii) Assume the parent of Leaf *n* is a reticulate node *y*_*i*_ in *N*_*i*_ for *i*=1,2. C-Rotate(*N*_1_,*x*_1_,*y*_1_) is identical to C-Rotate(*N*_2_,*x*_2_,*y*_2_) iff *N*_1_=*N*_2_.

#### *Proof*

(i) Let $N_{i} \in {\mathcal {T}CN}_{k}(n)$ and $e_{i}\in {\mathcal {V}}(N_{i}), i=1, 2$. Let *M*_1_=Leaf-Insert(*N*_1_,*e*_1_,*n*+1) and *M*_2_=Leaf-Insert(*N*_2_,*e*_2_,*n*+1) such that *M*_1_=*M*_2_. Then, there exists a node mapping *ϕ* from *M*_1_ to *M*_2_ such that (i) it maps a leaf in *M*_1_ to the same leaf and (ii) $(\phi (u), \phi (v))\in {\mathcal {E}}(M_{2})$ if and only if $(u, v)\in {\mathcal {E}}(M_{1})$. Since *n*+1 is inserted as a leaf, *ϕ* maps the parent *p*_1_ of (*n*+1) in *M*_1_ to the parent *p*_2_ of *n*+1 in *M*_2_, implying that *ϕ* induces an isomorphic mapping from *N*_1_ to *N*_2_. This proves the necessity condition. The sufficient condition is straightforward.

(ii) and (iii) Both statement can be proved similarly. The proposition is proved. □

**Fig. 4:**
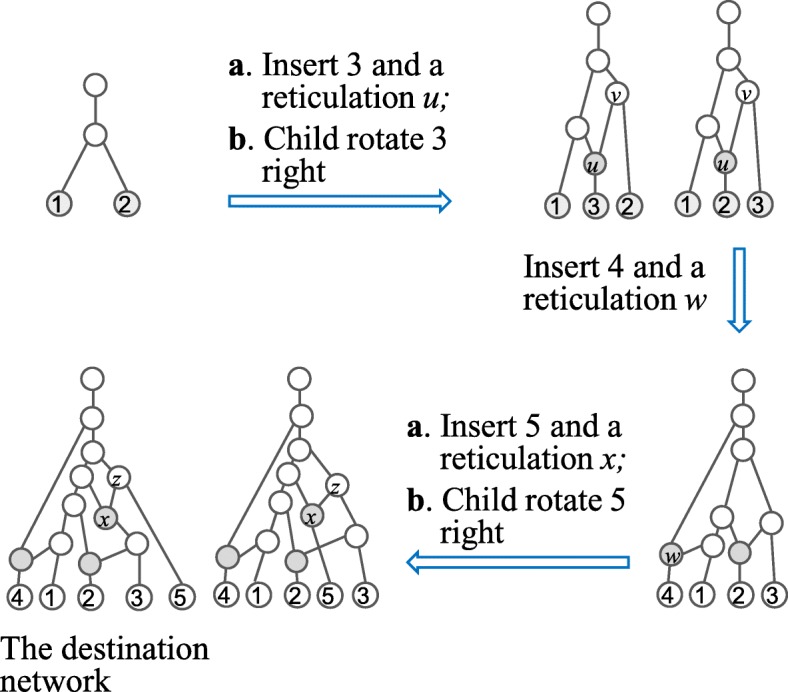
Illustration how to generate the left TCN in Figure 1 from a tree on 2 taxa

Figure 4 show how to generate the left TCN given in Fig. 1.

## Results

### Main theorems

Taken together, Propositions 1–4 imply the following theorem.

#### **Theorem 1**

Each TCN of ${\mathcal {T}CN}_{k+1}(n+1)$ can be obtained from either (i) a unique TCN of ${\mathcal {T}CN}_{k+1}(n)$ by attaching Leaf *n*+1 to a tree edge or (ii) a unique TCN $N\in {\mathcal {T}CN}_{k}(n)$ by applying one of the following operations:

- Insertion of a reticulate node *r* with the child Leaf (*n*+1) into a tree edge or straddling two tree edges;
- Insert *r* into a tree edge (*u*,*v*), as described in (a), and then conduct the child rotation to switch the child of *r* and the tree node child of *v*.
- Insert *r* straddling two tree edges *e*^′^=(*u*^′^,*v*^′^) and *e*^′′^=(*u*^′′^,*v*^′′^), as described in (a), and then conduct the child rotation to switch the child of *r* and the tree node child of *v*^′′^ (resp. *v*^′^) if *u*^′′^ (resp. *u*^′^) is not an ancestor of *u*^′^ (resp. *u*^′′^).

**Fig. 5:**
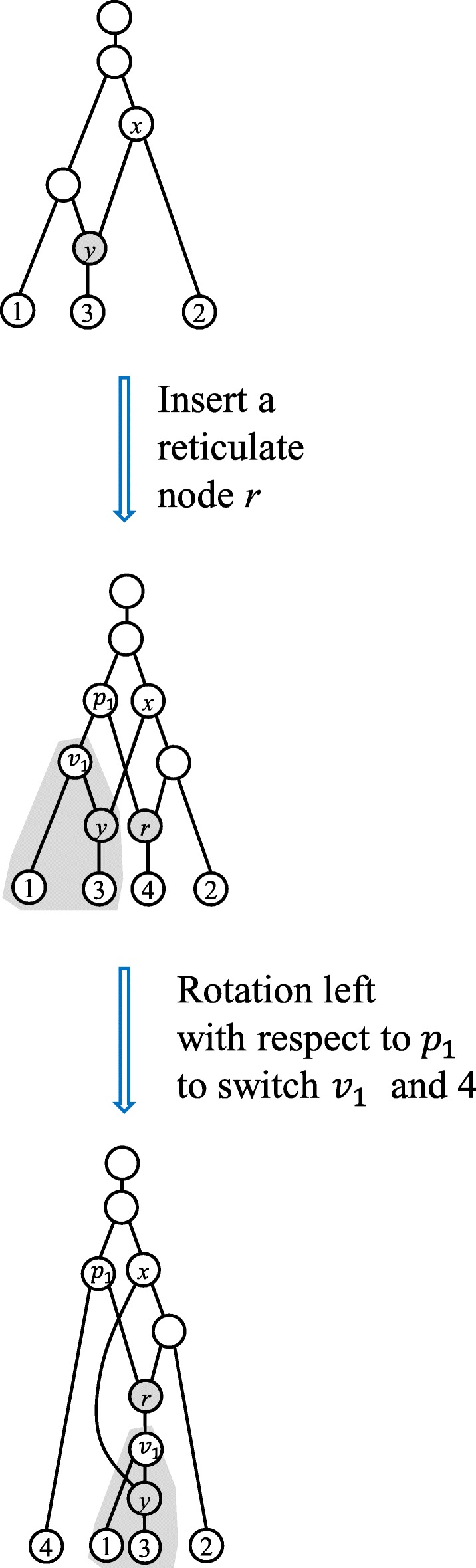
Illustration the undesired condition in Theorem 2 that prevents from applying s a child rotation. Here, the reticulate node *r* and its child Leaf 4 are first inserted into the tree edges entering *v*_1_ and *v*_2_(i.e. 2) in a normal network (top), generating a normal network (middle). But, child rotation to left leads to a tree-child network that is no longer normal (bottom), in which *y* does not satisfy the normal condition

If we restrict the operations on normal networks, we obtain all the normal networks in ${\mathcal {T}CN}_{k+1}(n+1)$. However, inserting a reticulate node and then applying the child rotation may lead to a scenario that a reticulation no longer satisfy the normal condition (Fig. 5). Hence, the child-rotation operation should be taken after some verification when all normal networks are enumerated.

#### **Theorem 2**

Each normal network of ${\mathcal {T}CN}_{k+1}(n+1)$ can be obtained from either (i) a unique normal network in ${\mathcal {T}CN}_{k+1}(n)$ by attaching Leaf *n*+1 to a tree edge or (ii) a unique normal network $N\in {\mathcal {T}CN}_{k}(n)$ by applying one of the following operations for each pair of incomparable edges *e*_1_=(*u*_1_,*v*_1_) and *e*_2_=(*u*_2_,*v*_2_) in *N*:

- Insert a reticulate node *r* with the child (*n*+1) straddling *e*_1_ and *e*_2_. Let *p*_*i*_ be the tree node inserted into *e*_*i*_ for *i*=1,2.
- Insert *r* as described in (a) and then conduct the child rotation to make *v*_1_ to be the child of *r* and *n*+1 the child of *p*_1_, respectively, unless a reticulate edge (*x*,*y*) exists in *N* (Fig. 5) such that:
  - *y* is below *v*_1_;
  - *x* is not an ancestor of *v*_1_;
  - *x* is an ancestor of *v*_2_.
- Insert *r* as described in (a) and then conduct the child rotation to make *v*_2_ to be the child of *r* and *n*+1 the child of *p*_2_, respectively, unless a reticulate edge (*x*,*y*) exists in *N* such that:
  - *y* is below *v*_2_;
  - *x* is not an ancestor of *v*_2_;
  - *x* is an ancestor of *v*_1_.

#### *Proof*

The statement for normal networks is based on the fact that if *N* is obtained from *N*^′^ vis one of the three operations given in Theorem 1, that the normality of *N* implies the normality of *N*^′^.

The conditions in (b) and (c) are used to exclude the child rotations that make the normal condition invalid for some existing reticulate nodes in the generated TCN. □

### Counting formulas

Let *N* be a TCN. For a pair of edges (*u*_1_,*v*_1_) and (*u*_2_,*v*_2_) of *N*, they are *incomparable* if neither of *v*_1_ and *v*_2_ is an ancestor of the other. Let *u*(*N*) be the number of unordered pairs of incomparable edges in *N* and let:

1 $$\begin{array}{@{}rcl@{}}  u_{n-1, k-1}=\sum_{N\in {\mathcal{T}CN}_{k-1}(n-1)} u(N). \end{array} $$

Define *a*_*n*,*k*_ to be $|{\mathcal {T}CN}_{k}(n)|, 0\leq k< n$ and *b*_*n*,*k*_ to be the number of normal networks in *TCN*_*k*_(*n*),0≤*k*<*n*.

#### **Theorem 3**

(i) The *a*_*n*,*k*_ can be calculated through the following recurrence formula:

2 $$\begin{array}{@{}rcl@{}} a_{n,k}&=& (2n+k-3)\{a_{n-1,k}+(2n+k-4)a_{n-1, k-1}\} \\ &&+u_{n-1, k-1},  \end{array} $$

where *a*_2,0_=1 and *u*_*n*−1,*k*−1_ is defined in Eq. (1).

(ii) The *b*_*n*,1_ can be calculated through the following recurrence formula:

3 $$\begin{array}{@{}rcl@{}}  {}b_{n, n-1}&=&0, \\ b_{n, 1}&=& (2n-2)b_{n-1, 1}+3u_{n-1, 0}\quad n>2, \end{array} $$

where *u*_*n*−1,0_ is the total number of unordered pairs of incomparable edges in all the phylogenetic trees on *n*−1 taxa.

#### *Proof*

(i) The unique tree on two taxa is a TCN and thus *a*_2,0_=1.

Each TCN of ${\mathcal {T}CN}_{k}(n-1)$ has 2*n*+*k*−3 tree edges and Leaf *n* can be attached to each of these edges. The first term of the right hand side of Eq. (2) counts the TCNs obtained by applying the leaf insertion in Theorem 1.

Consider $N\in {\mathcal {T}CN}_{k-1}(n-1)$. *N* has *n*−1 leaves, *n*+*k*−3 tree nodes, and thus 2*n*+*k*−4 tree edges. The reticulation insertion can be used on a single edge or a pair of edges in *N*. Thus, we can insert a reticulate node *r* with the child Leaf *n* in $2n+k-4 +{2n+k-4 \choose 2}=(2n+k-3)(2n+k-4)/2$ possible ways. After the insertion of *r* in a tree edge (*u*,*v*), we can apply a child rotation to exchange Leaf *n* with *v*, as *u* is not an ancestor of *v* after *r* was inserted. Similarly, after *r* is connected to a pair of edges *e*_1_ and *e*_2_, we can apply a child rotation once if one edge is below the other and in two possible ways if neither is an ancestor of the other.

In summary, for each unordered pair of tree edges (*e*_1_,*e*_2_), we can generate three different tree child networks with *k* reticulations on [1,*n*] if they are incomparable and two otherwise. Thus, we have the second and third terms of the formula.

(ii) The fact that *b*_*n*,*n*−1_=0 was first proved by Bickner [25]. □

In the case that *n*>2 and *k*≤*n*−2, Eq. (3) for *b*_*n*,1_ follows from the following two facts:

- Only two incomparable edges in normal networks in ${\mathcal {T}CN}_{k-1}(n-1)$ can be used to generate normal networks in ${\mathcal {T}CN}_{k}(n)$;
- For each unordered pair of incomparable edges in a tree on [1,*n*−1], three normal networks can be obtained by applying insertion of reticulate node and two child rotations.

Unfortunately, we do not know how to obtain a simple formula for *b*_*n*,*k*_ in general. By Theorem 3, one still can compute the number of normal networks with *k* reticulate nodes on [1,*n*],*b*_*n*,*k*_, by enumeration. For each 1≤*k*≤*n*−2 and 3≤*n*≤7,*b*_*n*,*k*_ is listed in Table 1.

**Table 1:** Counts of the normal networks with *k* reticulations on [*n*],1≤*k*≤*n*−2 and 3≤*n*≤7

*k*\*n*	3	4	5	6	7	8
1	3	54	855	14,040	248,535	4,787,370
2		48	2310	78,120	2,377,620	70,749,000
3			1920	184,680	11,038,530	536,524,830
4				146,520	23,797,302	2,217,404,379
5					16,198,764	3,802,965,091
6						2,479,006,101

It is challenging to obtain a simple formula for counting *u*_*n*,*k*_ for arbitrary *k*. But we can find a closed formula for *u*_*n*,0_ and thus obtain a recurrence formula for *a*_*n*,1_ and *b*_*n*,1_.

#### **Lemma 1**

For any *n*≥2, the total number of unordered pairs of incomparable edges in all the phylogenetic trees on *n* taxa is:

4 $$\begin{array}{@{}rcl@{}} u_{n, 0}=\frac{(n+1)(2n)!}{2^{n}(n)!} -2^{n}n!  \end{array} $$

#### *Proof*

Let *T* be a phylogenetic tree on [1,*n*−1] and let *O*(*T*) denote the set of ordered pairs of comparable edges in *T*, where that (*x*,*y*)∈*O*(*T*) means the edge *x* is above the edge *y*. Then, it is not hard to verify:

$$\begin{array}{@{}rcl@{}} {}O(T)&=&\cup_{e\in {\mathcal{E}}(T)} \{(e, x) \;|\; x\in {\mathcal{E}}(T) \text{ s.t. \text{e} is above \text{x}}\}\\ &=&\cup_{e\in {\mathcal{E}}(T)} \{(y, e) \;|\; y\in {\mathcal{E}}(T) \text{ s.t. \text{e} is below \text{y}}\} \end{array} $$

□

Assume *T*^′^ is obtained from *T* by attaching Leaf *n* in an edge *e*=(*u*,*v*). In *T*^′^, the parent *w* of Leaf *n* is the tree node inserted in *e*, implying that *e* is subdivided into two edges of *T*^′^:

$$e_{1}=(u, w), \;\;e_{2}=(w, v),$$

and

$${\mathcal{E}}(T')=\{e_{1}, e_{2}, (w, n)\}\cup {\mathcal{E}}(T) - \{e\}. $$

Thus,

$$\begin{array}{@{}rcl@{}} O(T') &=& \{(e_{1}, e_{2}), (e_{1}, (w, n))\} \\ && \cup \{(x, y) \in O(T) \;|\; x\neq y, x\neq e \neq y \}\\ & & \cup \{ (e', e_{1}), (e', e_{2}), (e', (w, n)) \;|\; (e', e)\in O(T)\} \\ & & \cup \{(e_{1}, e^{\prime\prime}), (e_{2}, e^{\prime\prime}) \;|\; (e, e^{\prime\prime})\in O(T)\}. \end{array} $$

Hence,

$$\begin{array}{@{}rcl@{}} && |O(T')|\\ &= &|O(T)|+2+2|\{(x, e) \;|\; x\in {\mathcal{E}}(T): (x, e)\in O(T) \}|\\ && + |\{(e, y) \;|\; y\in {\mathcal{E}}(T) \text{ s.t. \text{y} is below \text{e}}\}|. \end{array} $$

Since *T* has 2*n*−3 edges,

5 $$\begin{array}{@{}rcl@{}} {} \sum_{T'\in \mathcal{LI}(T, n)}|O(T')| &=&(|{\mathcal{E}}(T)|+3) |O(T)|+2 |{\mathcal{E}}(T)|  \\ &=&2n\times O(T)+2(2n-3).  \end{array} $$

where $\mathcal {LI}(T, n)$ denotes the set of 2*n*−3 phylogenetic trees that are obtained by a Leaf-Insertion on *T*.

Let *c*_*n*_ be the total number of unordered pairs of comparable edges in all the phylogenetic trees on *n* taxa. Clearly, *c*_2_=2. Since there are $\frac {(2n-4)!}{2^{n-2}(n-2)!}$ phylogenetic trees with *n*−1 leaves, which each have 2*n*−3 edges, Eq. (5) implies:

$$\begin{array}{@{}rcl@{}} c_{n}&=&2nc_{n-1}+\frac{2(2n-2)!}{2^{n-1}(n-1)!} \end{array} $$

or, equivalently,

$$\begin{array}{@{}rcl@{}} \frac{1}{n!}c_{n}&=&2\left(\frac{1}{(n-1)!}c_{n-1}\right)+\frac{(2n-2)!}{2^{n-2}n!(n-1)!}. \end{array} $$

Therefore,

$$\begin{array}{@{}rcl@{}} c_{n} &=& {n!2^{n}} \sum^{n-1}_{k=1} \frac{1}{(k+1)!}\frac{(2k)!}{2^{2k}(k)!}\\ &=& \frac{n!2^{n-1}}{\pi} \sum^{n-1}_{k=1} \int^{4}_{0}\left(\frac{x}{4}\right)^{k}\left(\frac{4-x}{x}\right)^{1/2}dx\\ &=& \frac{n!2^{n+1}}{\pi} \int^{1}_{0} (1-x^{n-1})\left(\frac{x}{1-x}\right)^{1/2}dx\\ &=& 2^{n} n! - \frac{(2n)!}{2^{n-1}n!}, \end{array} $$

where $\frac {(2k)!}{(k+1)!k!}$ is the *k*-th Catalan number *C*_*k*_ that is equal to the integral appearing above ([31]). Since there are $\frac {(2n-2)!}{2^{n-1}(n-1)!}$ phylogenetic trees on [1,*n*] each having (2*n*−1) edges,

$$\begin{array}{@{}rcl@{}} u_{n, 0}&=&\frac{(2n-2)!}{2^{n-1}(n-1)!} {2n-1 \choose 2} -c_{n}\\ & = & \frac{(2n-2)!}{2^{n-1}(n-1)!} (n-1)(2n-1) + \frac{(2n)!}{2^{n-1}n!} - 2^{n} n! \\ & = & \frac{(n+1)(2n)!}{2^{n}n!} -2^{n} n!. \end{array} $$

#### **Theorem 4**

For any *n*≥3, the numbers of TCNs and normal networks with exactly one reticulate node on *n* taxa are:

6 $$\begin{array}{@{}rcl@{}}  a_{n, 1} &= & \frac{(2n)!}{2^{n} (n-1)!}-2^{n-1} n! \end{array} $$

and

7 $$\begin{array}{@{}rcl@{}}  b_{n, 1} =\frac{(n+2)(2n)!}{2^{n}n!}-3\cdot 2^{n-1}n!, \end{array} $$

respectively.

#### *Proof*

Since $a_{n-1, 0}=\frac {(2n-4)!}{2^{n-2}(n-2)!}$, by Theorem 3,

$$\begin{array}{@{}rcl@{}} a_{n, 1}=2(n-1)a_{n-1, 1}+\frac{(3n-2)(2n-3)!}{2^{n-2}(n-2)!}-2^{n-1}(n-1)! \end{array} $$

or, equivalently,

$$\begin{array}{@{}rcl@{}} \frac{a_{n, 1}}{(n-1)!}=2\left(\frac{a_{n-1, 1}}{(n-2)!}\right)+\frac{(3n-2)(2n-2)!}{2^{n-1}((n-1)!)^{2}}-2^{n-1} \end{array} $$

Therefore, since *a*_2,1_=2,

$$\begin{aligned} \frac{a_{n, 1}}{(n-1)!}&\\ &= 2^{n-2} \left(\frac{a_{2, 1}}{(2-1)!}\right)\\ &\quad+ \sum^{n-2}_{i=1} \frac{(3n-3i+1)(2n-2i)!}{2^{n-2i+1}((n-i)!)^{2}} - 2^{n-1} (n-2) \\ &= \sum^{n-2}_{i=1} \frac{(3n-3i+1)(2n-2i)!}{2^{n-2i+1}((n-i)!)^{2}} - 2^{n-1} (n-3) \\ &= 2^{n-1}\sum^{n-2}_{i=1} \frac{(3(n-i)+1)(2(n-i))!}{2^{2(n-i)}((n-i)!)^{2}} - 2^{n-1} (n-3) \\ &= 2^{n-1}\sum^{n-1}_{k=2} \frac{(3k+1)(2k)!}{2^{2k}(k!)^{2}} - 2^{n-1} (n-3) \\ &= 2^{n-1}\sum^{n-1}_{k=1} {2k\choose k} \frac{3k+1}{4^{k}} - 2^{n-1} (n-1). \end{aligned} $$

By induction, we can show that $\sum ^{n}_{k=0}{2k\choose k}4^{-k}=\frac {(2n+1)!}{2^{2n}(n!)^{2}}$ and $\sum ^{n}_{k=0}{2k\choose k}k4^{-k}=\frac {(2n+1)!}{3\cdot 2^{2n}n!(n-1)!}$. Continuing the above calculation, we obtain:

$${}\frac{a_{n, 1}}{(n-1)!}=\left\{\frac{(2n-1)!}{2^{(n-1)}(n-1)!}\left[\frac{1}{(n-2)!}+\frac{1}{(n-1)!}\right]-n\right\}.$$

□

This proves Eq. (6).

Similarly, by Theorem 3 and Lemma 1, we have:

$$\begin{array}{@{}rcl@{}} b_{n, 1}&=& 2(n-1)b_{n-1, 1} \\ & &+ 3\cdot \left(\frac{n(2n-3)!}{2^{n-2}(n-2)!}-2^{n-1}(n-1)!\right)\\ \end{array} $$

or, equivalently,

$$\begin{array}{@{}rcl@{}} \frac{b_{n, 1}}{(n-1)!}&=& 2\frac{b_{n-1, 1}}{(n-2)!} + 3 {2n-3\choose n-1}\frac{n}{2^{n-2}}-3\cdot 2^{n-1}. \end{array} $$

Since *b*_2,1_=0,

$$\begin{array}{@{}rcl@{}} &&\frac{b_{n, 1}}{(n-1)!}\\ &=& 2^{n-2}\frac{b_{2, 1}}{(2-1)!}\\ && +3\cdot 2^{n} \cdot \sum^{n-2}_{i=1} {2n-2i-1\choose n-i}\frac{n-i+1}{2^{2n-2i}}\\ && -3\cdot 2^{n-1} (n-2)\\ &=& 3\cdot 2^{n} \cdot \sum^{n-1}_{k=1} {2k-1\choose k}\frac{k+1}{2^{2k}}-3\cdot 2^{n-1} (n-1)\\ &=& 3\cdot 2^{n-1} \cdot \sum^{n-1}_{k=1} {2k \choose k}\frac{k+1}{2^{2k}}-3\cdot 2^{n-1} (n-1)\\ &=&\frac{(n+2)(2n)!}{2^{n}n!}-3\cdot 2^{n-1}n!. \end{array} $$

This proves Eq. (7).

#### **Remark 1**

Every RPN with exactly one reticulate node is a TCN. Therefore, *a*_*n*,1_ is actually the number of RPNs with one reticulate node.

## Conclusions

It is well-known that all phylogenetic trees on *n* taxa can be generated by the insertion of the *n*-th taxa in each edge of all the phylogenetic trees on the first *n*−1 taxa. The main result of this work is a generalization of this fact into TCNs. This leads to a simple procedure for enumerating both normal networks and TCNs, the C-code for which is available upon request. It is fast enough to count all the normal networks on eight taxa. Recently, Cardona et al. introduced a novel operation to enumerate TCNs. Their program was successfully used to compute the exact number of tree-child networks on six taxa. Although our program is faster than theirs, it still cannot be used to count TCNs on eight taxa on a PC.

Another contribution of this work is Eq. (6) and (7) for counting RPNs with exactly one reticulate node. Semple and Steel [30] presented formulas for counting unrooted networks with one reticulate node. Since an unrooted networks can be oriented into a different number of RPNs, it is note clear how to use their results to derive a formula for the count of RPNs. Bouvel et al. [26] presented a formula for counting RPNs with one reticulate node. Our formula is much simpler than the formula given in [26].

Lastly, the following problem is open:

Is there a simple formula like Eq. (6) for the count of TCNs with *k* reticulate nodes on *n* taxa for each *k*>1?

## Data Availability

Data sharing is not applicable to this article as no datasets were generated or analysed during the current study.

## Abbreviations

NNI: Nearest neighbor interchange
RPN: Rooted phylogenetic network
TCN: Tree-child network
